# A Canadian evaluation framework for quality improvement in childhood arthritis: key performance indicators of the process of care

**DOI:** 10.1186/s13075-020-02151-w

**Published:** 2020-03-19

**Authors:** Claire E. H. Barber, Marinka Twilt, Tram Pham, Gillian R. Currie, Susanne Benseler, Rae S. M. Yeung, Michelle Batthish, Nicholas Blanchette, Jaime Guzman, Bianca Lang, Claire LeBlanc, Deborah M. Levy, Christine O’Brien, Heinrike Schmeling, Gordon Soon, Lynn Spiegel, Kristi Whitney, Deborah A. Marshall

**Affiliations:** 1 Department of Medicine, Cumming School of Medicine, University of Calgary, Calgary, AB Canada; 2 Department of Community Health Sciences, Cumming School of Medicine, University of Calgary, Calgary, AB Canada; 3 Department of Pediatrics, Alberta Children’s Hospital, Cumming School of Medicine, and Alberta Children’s Hospital Research Institute, University of Calgary, Calgary, AB Canada; 4 Departments of Pediatrics, Immunology and Medical Science, University of Toronto, Toronto, ON Canada; 5 The Hospital for Sick Children, Toronto, ON Canada; 6 Department of Pediatrics, McMaster University and McMaster Children’s Hospital, Hamilton, ON Canada; 7 Trillium Health Partners, Mississauga, ON Canada; 8 Department of Medicine, University of British Columbia, Vancouver, BC Canada; 9 Department of Pediatrics, Dalhousie University, Halifax, NS Canada; 10 Department of Pediatrics, McGill University, Montreal, QC Canada

**Keywords:** Juvenile idiopathic arthritis, Quality improvement, Quality of care, Quality indicators

## Abstract

**Background:**

The evaluation of quality of care in juvenile idiopathic arthritis (JIA) is critical for advancing patient outcomes but is not currently part of routine care across all centers in Canada. The study objective is to review the current landscape of JIA quality measures and use expert panel consensus to define key performance indicators (KPIs) that are important and feasible to collect for routine monitoring in JIA care in Canada.

**Methods:**

Thirty-seven candidate KPIs identified from a systematic review were reviewed for inclusion by a working group including 3 pediatric rheumatologists. A shortlist of 14 KPIs was then assessed using a 3-round modified Delphi panel based on the RAND/UCLA Appropriateness Method. Ten panelists across Canada participated based on their expertise in JIA, quality measurement, or lived experience as a parent of a child with JIA. During rounds 1 and 3, panelists rated each KPI on a 1–9 Likert scale on themes of importance, feasibility, and priority. In round 2, panelists participated in a moderated in-person discussion that resulted in minor modifications to some KPIs. KPIs with median scores of ≥ 7 on all 3 questions without disagreement were included in the framework.

**Results:**

Ten KPIs met the criteria for inclusion after round 3. Five KPIs addressed patient assessments: pain, joint count, functional status, global assessment of disease activity, and the clinical Juvenile Arthritis Disease Activity Score (cJADAS). Three KPIs examined access to care: wait times for consultation, access to pediatric rheumatologists within 1 year of diagnosis, and frequency of clinical follow-up. Safety was addressed through KPIs on tuberculous screening and laboratory monitoring. KPIs examining functional status using the Childhood Health Assessment Questionnaire (CHAQ), quality of life, uveitis, and patient satisfaction were excluded due to concerns about feasibility of measurement.

**Conclusions:**

The proposed KPIs build upon existing KPIs and address important processes of care that should be measured to improve the quality of JIA care. The feasibility of capturing these measures will be tested in various data sources including the Understanding Childhood Arthritis Network (UCAN) studies. Subsequent work should focus on development of meaningful outcome KPIs to drive JIA quality improvement in Canada and beyond.

## Introduction

Juvenile idiopathic arthritis (JIA) is an inflammatory arthritis that affects approximately 3 in 1000 Canadian children [1]. Timely diagnosis and treatment in a pediatric rheumatology center is key to improving outcomes of patients with JIA. While contemporary treatments have resulted in excellent outcomes for many patients, there can be variability in outcomes with a severe disease course in about 20% of patients in contemporary cohorts [2]. For some with JIA, disease may persist into adulthood [3–5]. In a Norwegian study, after 30 years of follow-up, persistence of active disease or medication use was seen in 41% of patients with JIA, with up to 28% having a high symptom state [6]. Consequences of inadequately treated disease include pain, functional limitations due to joint deformities or damage, growth abnormalities, and psychological impacts [7, 8]. Beyond the burden on patients and families, JIA can also be a costly disease. Patients who fail first-line disease-modifying anti-rheumatic drugs (DMARDs) are often treated with biologic therapies, which are highly effective in improving outcomes, but are costly [9]. Indeed, tumor necrosis factor inhibitors (anti-TNF), the biologic DMARDs most often used in JIA, rheumatoid arthritis (RA), and inflammatory bowel disease, are the drug class that accounted for the highest proportion of public drug spending in Canada in 2018 [9].

Variability in practice and care can contribute to suboptimal patient outcomes. Over the last number of decades, there has been increasing interest in measuring and monitoring processes and outcomes of care to reduce unwarranted variability and improve quality of care, defined as “the degree to which health services for individuals and populations increase the likelihood of desired health outcomes and are consistent with current professional knowledge” [10]. In the USA, the American College of Rheumatology (ACR) published a White Paper on Quality Measurement in 2011 highlighting the quality landscape for rheumatologists and prioritizing areas for measure development [11]. In that White Paper, JIA ranked 3rd as an area for future ACR quality measure development following RA and osteoporosis. Despite this, there currently exist no ACR-endorsed JIA quality measures [12]. Nevertheless, groups such as the Pediatric Rheumatology Care and Outcomes Improvement Network (PR-COIN) in the USA and the British Society for Pediatric and Adolescent Rheumatology (BSPAR) in the UK have proposed measurement tools for quality improvement in JIA [13, 14]. In Canada, the Arthritis Alliance of Canada (AAC), a non-profit group representing 36 arthritis stakeholders’ groups, developed a set of system-level performance measures for inflammatory arthritis including JIA [15]. The measures focused on early access to care and treatment, and 3 of the measures were applicable to JIA. However, the measures addressed only wait times, yearly follow-up, and pediatric rheumatology access within 1 year [15].

The objective of this study was to review the current landscape of JIA quality measures and through expert panel consensus to define key performance indicators (KPIs) that are important and feasible to collect for routine monitoring of JIA care in Canada. The KPIs will be implemented and tested in future studies and will help assess strategies for care improvement. The KPI framework also represents a first step in the evaluation of individual patient and health economic outcomes.

## Methods

**Fig. 1:**
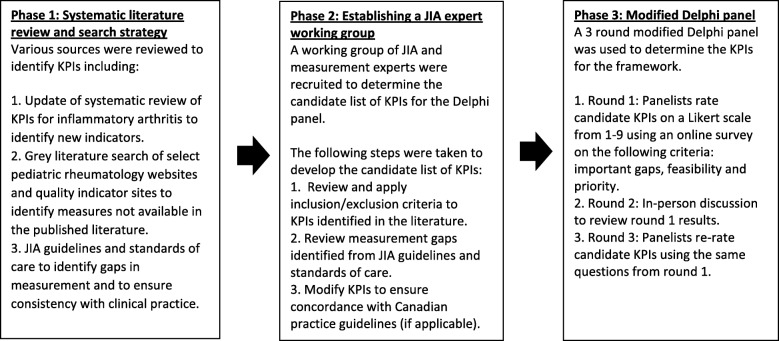
Summary of key performance indicators development process

The evaluation framework was developed over 3 phases using a modification of the RAND Corporation/University of California Los Angeles/University of California Los Angeles (RAND/UCLA) Appropriateness Method [16]. A summary of this process is shown in Fig. 1.

### Phase 1: Systematic literature review and search strategy

A systematic search of KPIs for inflammatory arthritis including JIA that has previously been published was updated to ensure the framework was aligned with existing KPIs, quality improvement efforts, and clinical practice guidelines [17]. To identify any new indicators that were developed since the original search, the search strategy was updated in January 2019. A grey literature search was also repeated of select websites (see Additional file 1) to identify any indicators not available in the peer-reviewed literature. To ensure appropriate scope and relevance, the grey literature search was limited to websites that pertained to pediatric rheumatology organizations and organizations that develop and report on quality indicators from Canada, the USA, and the UK. Experts in the field from the PR-COIN network and Canadian Rheumatology Association Quality of Care Committee and the American College of Rheumatology Quality Measures sub-committees were also contacted about unpublished quality measurement frameworks, and the reference lists of included articles were searched for additional sources.

Existing KPI publications were included in our updated review if (1) they were written in English, (2) identified an indicator of JIA quality of care, and (3) the method of indicator development was available. KPIs were excluded if there was no description of how they were selected and/or developed.

JIA guidelines and standards of care were identified using a separate targeted search strategy of pediatric rheumatology organizations in Canada and internationally (see Additional file 2). The guidelines and standards of care were used to (1) identify gaps in measurement when compared to the existing KPIs identified from the systematic review and (2) to ensure that the identified KPIs were supported by established clinical practice guidelines.

### Phase 2: Establishing a JIA expert working group

A working group was assembled to guide and oversee the development of the evaluation framework (CB, DM, MT, LS, and NB). Working group members were recruited based on their clinical expertise with JIA (MT, LS, and NB) and/or performance measurement (CB, DM, and MT). No individuals declined the invitation to participate. Members were asked to participate in a series of conference calls to review the project methodology and draft KPIs before presentation to the modified Delphi panel.

Several steps were taken to develop the candidate list of KPIs for the modified Delphi panel (Fig. 1). Working group members reviewed the 37 KPIs identified from the systematic review separately, and then members convened as a group to discuss the exclusion criteria (Table 1). Two members of the team (CB and TP) consolidated the working group feedback to determine the shortlist of candidate KPIs to be discussed by the Delphi panel.

**Table 1:** Exclusion criteria for the candidate key performance indicators for the modified Delphi panel

1. Indicator covers a low priority area*	
2. Indicator estimation too complex (i.e., unlikely to be feasible to measure)	
3. Indicator similar to existing AAC System-Level Performance Measure, suggested we use AAC measure	
4. Indicator covers a concept addressed in other measures but is less clearly defined than included measure	
5. Indicator specific to nursing (highly specific to nurse-led models of care)	
6. Indicator covers a similar concept covered in an already-included measure	
7. Indicator specific to physiotherapy (not clearly pediatric rheumatology care)	
8. Indicator does not align with current JIA guidelines	
9. Indicator does not meet Canadian benchmarks^+^	
10. Indicator is not under the control of pediatric rheumatologists (i.e., depends on other health care specialist or provider)	

*AAC* Arthritis Alliance of Canada

*Low priority areas were determined by the working group members

^+^Benchmarks based on the Canadian Rheumatology association wait time benchmarks for arthritis care [18]

Following this process, the working group members were asked to review measurement gaps that were identified when comparing the KPIs to the established guidelines and standards of care for JIA. At this stage, there were a few noted overlaps between KPIs, and additional measures were proposed by the working group to address this issue. In addition, slight modifications to the wording and/or specification of some KPIs were made to ensure concordance with Canadian practice guidelines and standards of care.

### Phase 3: Modified Delphi panel

#### Panelist recruitment

Fourteen JIA stakeholders including 9 pediatric rheumatologists, 2 allied health professionals, and 3 parents of a child with JIA were invited to participate in the modified Delphi panel to select the KPIs in the evaluation framework. Participants are part of the larger Understanding Childhood Arthritis Network (UCAN) CURE team as either collaborators or as part of the patient engagement committee and were selected based on their clinical background as pediatric rheumatologists caring for patients with JIA, professional expertise as allied health professionals in pediatric rheumatology, or personal experience with JIA. To ensure diversity of representation, panelists were recruited from various centers across Canada. Participants did not receive any honoraria or incentives for their participation in the study. The University of Calgary Conjoint Health Research Ethics Board approved this study (REB19-0098).

#### Modified Delphi panel protocol

The modified Delphi panel consisted of 3 rounds, including 2 rounds of voting using an online survey with an in-person panel discussion in between. In round 1, the modified Delphi panel rated each KPI on a Likert scale from 1 to 9 using the following criteria: (1) does the measure target an important gap in JIA care, (2) how likely is it that the information required to report this measure will be available to health care providers in a typical pediatric rheumatology clinic, 3) overall priority of including this indicator in the evaluation framework. The round 1 survey also included basic demographic questions such as the number of years in practice for health professionals. Panelists were given a background document of the study rationale, methodology, supporting guideline summary, and measurement specifications for reference during the voting process. In round 2, panelists participated in an in-person meeting to review the results from round 1 voting and to discuss any concerns. In round 3, panelists re-rated the KPIs using the same criterion questions as in round 1.

#### Analysis of panelist responses

Panel ratings for each criterion were categorized into “high” (median scores of 7–9), “uncertain” (median scores of 4–6), and “low” (median scores of 1–3). Median scores of 3.5 or 6.5 were included in the next higher rating category as recommended for panels comprised of an even number of panelists [16]. To be included in the evaluation framework, KPIs had to have median scores of ≥ 7 on all 3 questions with no disagreement among panelists. Disagreement was calculated using the method outlined by the RAND/UCLA Manual [16] and exists when the interpercentile range (difference between the 30th and 70th percentiles) is larger than the Interpercentile Range Adjusted for Symmetry (IPRAS), which was calculated using the following formula: IPRAS = 2.35 + (Asymmetry Index × 1.5).

## Results

### Phase 1: Systematic literature review and search strategy

The systematic literature search identified 276 articles, of which 9 were reviewed in full text. For consideration in this phase of framework development, only one [19] was included from the update of the search (see Additional file 1 for flow diagram), in addition to two other publications previously identified [15, 20]. Thirty-seven KPIs were abstracted from the 3 articles and categorized into clinically-relevant themes (e.g., assessment, medication). To understand the breadth and depth of the KPIs, they were further classified as structure, process, or outcome indicators based on the Donabedian’s framework [21] and the dimensions of quality according to the Institute of Medicine (IOM) [10], and the Alberta Health Quality Matrix which is based on the IOM framework [22]. Lastly, the indicators were classified according to the AAC Pan-Canadian Model of Care (MOC) (e.g., specialized access to care, medical management) [23]. Several gaps in measures were found when comparing the abstracted KPIs to JIA guidelines and standards of care. These gaps mainly pertained to the use of non-drug therapies, transitional care, therapy assessment, and vaccinations.

### Phase 2: Establishing a JIA expert working group

**Fig. 2:**
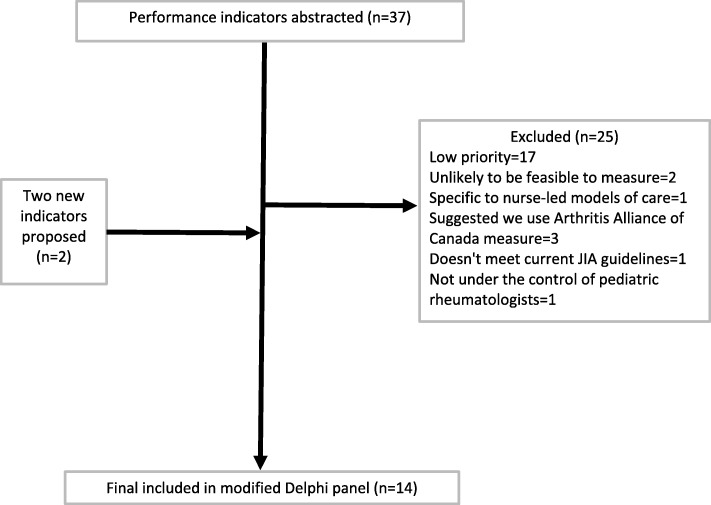
Flow diagram of key performance indicators included in modified Delphi panel

The working group members reviewed the draft KPIs, and a total of 14 candidate KPIs were included after an iterative review process (see Fig. 2). A brief summary of the recommendations and minor wording and specification changes made by the working group members are outlined in Additional file 3. Lastly, the working group members reviewed the measurement gaps (see Additional file 4). The consensus was that these areas of JIA care were challenging to measure and not feasible to assess given the scope and overall objectives of the evaluation framework.

### Phase 3: Modified Delphi panel

Of the 14 individuals approached, 10 agreed to participate in the modified Delphi panel. The panel consisted of 7 pediatric rheumatologists, 2 allied health professionals, and 1 parent of a child with JIA. There was representation from various provinces across Canada including Alberta, British Columbia, Nova Scotia, Ontario, and Quebec. The number of years in practice for health care providers ranged from 5 to 30 years. All panelists completed rounds 1 and 3 of the modified Delphi panel. Nine panelists participated in the in-person discussion. The panelist who could not participate in the in-person discussion reviewed detailed notes of the meeting and participated in round 3.

After round 1, several KPIs (KPIs 5, 10, 12, 13, and 14) scored in the uncertain range (median score of 4–6) on the feasibility criterion (without disagreement), while other KPIs were rated high (median score of 7–9) on all 3 rating questions. Following round 2 discussions, minor modifications were made to the wording or specification of some of the KPIs based on the feedback from the panelists. These changes are summarized in Additional file 3. After round 3 voting, 10 KPIs met the threshold for inclusion and 4 KPIs were excluded. The results of the panel voting from round 3 and the final included KPIs are shown in Table 2 and Table 3 respectively. The descriptions and reporting of the KPIs are shown in Additional file 5, and the complete specification of each KPI is available upon request.

**Table 2:** Results of modified Delphi panel (round 3)

KPI	Median (range) of 10 panel participants		
	Importance	Feasibility	Priority
**Indicator 1**: Assessment of arthritis-related pain	8 (6–9)	8 (6–9)	8 (8–9)
**Indicator 2**: Rheumatological joint count	9 (7–9)	8 (7–9)	9 (8–9)
**Indicator 3**: Physician’s global assessment of disease activity	8 (6–9)	7.5 (6–8)	8 (7–9)
**Indicator 4**: Assessment of functional ability	7 (5–9)	7 (3–8)	7 (6–9)
**Indicator 5:** Assessment of functional ability using the CHAQ	7 (3–9)	5.5 (2–8)	7 (4–9)
**Indicator 6**: Composite disease activity measurement	8 (6–9)	7 (5–8)	8 (6–9)
**Indicator 7**: Tuberculosis screening	8 (5–9)	8 (7–9)	8 (5–9)
**Indicator 8**: Laboratory monitoring for DMARDs	8 (6–9)	8 (7–9)	8 (7–9)
**Indicator 9**: Waiting times for rheumatologist consultation for patients with new onset JIA	8 (6–9)	7 (4–8)	8 (5–9)
**Indicator 10**: Percentage of patients with JIA seen by a rheumatologist	7.5 (6–9)	7 (1–9)	7 (4–9)
**Indicator 11**: Percentage of patients seen in yearly follow-up by a pediatric rheumatologist	7 (4–8)	8 (5–9)	7 (5–8)
**Indicator 12**: Median time from the patient’s first clinic visit to the date of their first uveitis screening	8 (6–9)	6 (2–9)	8 (3–9)
**Indicator 13**: Assessment of health-related quality of life	7 (4–8)	6 (2–8)	7 (5–8)
**Indicator 14**: Assessment of patients/parent satisfaction with care	7 (3–8)	5 (2–7)	7 (3–7)

*KPI* key performance indicator, *CHAQ* The Childhood Health Assessment Questionnaire, *DMARDs* disease-modifying anti-rheumatic drugs. Only KPIs with median scores of ≥ 7 on all 3 questions with no disagreement were included in the final set. Ratings 4–6 were categorized as “uncertain”

**Table 3:** Final set of key performance indicators

KPI name	Reporting during measurement period
Assessment of arthritis-related pain	% of patients assessed for pain at the first visit and each subsequent visit using any validated age-appropriate tool to measure average pain.
Rheumatological joint count	% of patients where a joint count was conducted on the first visit and each subsequent visit using a validated tool.
Physician’s global assessment of disease activity	% of patients assessed for a PGA using any validated tool at the first visit and at each subsequent visit.
Assessment of functional ability	% of patients assessed for functional ability using any validated tool at the first visit and at every routine clinic visit.
Composite disease activity measurement	% of patients in with an assessment of disease activity using the cJADAS.
Tuberculosis screening	% of patients screened for TB within 12 months prior to receiving a first course of therapy using a biologic DMARD.
Laboratory monitoring for DMARDs	% of patients who received methotrexate and leflunomide and monitored for toxicity by clinical laboratory methods.
Waiting times for rheumatologist consultation for patients with new onset JIA	The 50th and 90th percentile waiting times for rheumatologic consultation.
Percentage of patients with JIA seen by a rheumatologist	% of patients with new onset JIA (incident JIA) with at least 1 visit to a pediatric rheumatologist in the first year of diagnosis.
Percentage of patients seen in yearly follow-up by a pediatric rheumatologist	% of patients with JIA seen by their pediatric rheumatologist at least once every year over.

*KPI* key performance indicator, *PGA* Physician’s global assessment, *cJADAS* Clinical Juvenile Arthritis Disease Activity Score, *DMARDs* disease-modifying anti-rheumatic drugs, *TB* tuberculosis screening

The final set of KPIs examines important processes of JIA care including access to care and the measurement of patient outcomes (Table 3). Panelists voted in KPIs that assessed pain, joint count assessments, functional status, global assessment of disease activity, and the clinical Juvenile Arthritis Disease Activity Score (cJADAS). KPIs for wait time consultation, access to pediatric rheumatologist within 1 year of diagnosis, and follow-up care were included. Safety was addressed through tuberculous screening and laboratory monitoring KPIs. Although KPIs examining functional status using the Childhood Health Assessment Questionnaire (CHAQ) and quality of life were deemed important, there were concerns about the availability of this information in usual clinical practice, leading to the exclusion of these KPIs. Concerns regarding feasibility of measurement also led to the exclusion of KPIs related to uveitis and patient satisfaction.

## Discussion

The present study identified 10 KPIs that examine important processes of care for JIA that will be tested in future studies. This work builds upon previous national efforts to develop a measurement framework for monitoring and improving care for patients with inflammatory arthritis that was developed in collaboration with the AAC [15]. The AAC System-Level Performance Measure set included 6 measures that have been used to evaluate early access to care and treatment for inflammatory arthritis, including JIA [24–28]. All 3 of the measures previously included in the AAC measurement set (wait times for JIA care, percentage of patients seen within 1 year of diagnosis, and percentage of patients seen in yearly follow-up) were retained in the current measurement set. While many of these measures build upon existing published measures, some modifications were made to better reflect Canadian practice and/or current guidelines. Furthermore, a new measure was proposed to ensure measurement of disease activity with the cJADAS to optimize efforts in treating JIA to target [29].

Internationally, this work is complementary to a number of national and international efforts to monitor and improve the quality of care for patients living with JIA. For example, PR-COIN is a quality improvement learning network that uses patient registry data to inform quality improvement strategies to optimize processes of care and patient outcomes and includes 20 centers in the USA and Canada [30]. Beyond measurement of processes and outcomes, PR-COIN uses a number of strategies to improve care including pre-visit planning, strategies and toolkits for self-management, and patient/parent engagement [30]. PR-COIN’s quality measurement framework includes several process and outcome measures adapted from a set of 12 measures assessing processes of arthritis care that were also considered in the present study [20]. They were developed by a working group including representatives from the ACR, American Academy of Pediatrics, American Board of Pediatrics, and the Association of Rheumatology Health Professionals [20]. PR-COIN uses sophisticated strategies for audit and feedback based on their measurement framework and improvement science methods to drive change. Importantly, the network sites have been mapping captured data to electronic records to facilitate data entry and automate reporting.

In 2010, the BSPAR developed standards of care for children and young people with JIA that addressed the empowerment of patients, identification of JIA, referral to pediatric rheumatology care, access to a pediatric rheumatology multidisciplinary team, eye screening, access to treatment including joint injections, regular review to target “tight” control strategies, clinical networks and arrangements for shared care, and the care of adolescent patients [31]. An audit in 10 pediatric rheumatology centers using retrospective chart review demonstrated variable adherence to the standards of care including delays in access to care [32]. The audit highlighted a need for consensus on measurable JIA quality indicators, which prompted the development of the BSPAR National Audit Tool for JIA which was funded by the Health Care Quality Improvement Partnership, an independent organization that aims to promote quality in healthcare [19, 33]. The audit tool contains not only quality measures but also prospectively collected patient and care giver reported outcome measures and experience measures (PROMs and PREMs) in a questionnaire. In parallel, a core dataset for JIA was developed with the vision of collecting standardized items in the audit tool to facilitate quality improvement and research efforts [19]. In the present study, items from the BSPAR audit tool were considered for inclusion in the framework; however, none were included in the final set of KPIs.

While our study represents the first Canadian national effort to define KPIs that are feasible and important to measure to drive improvements in processes of care in JIA, the ultimate goal is to improve patient outcomes. We did not, however, include any outcome KPIs in our framework at the present time for several reasons. Firstly, we relied on published quality measures as a starting point for our framework to ensure measures were comparable with other centers. At the time of our study, outcome measures from PR-COIN were not published and available in the public domain for consideration of inclusion [20]. The outcome measures reported from the BSPAR audit tool relied heavily on patient experience or outcome questionnaires, which were deemed less feasible to collect in our healthcare setting. Secondly, agreeing upon and monitoring of the process measures was considered a first step to the evaluation of outcomes and setting benchmarks. Following our study completion, the American College of Rheumatology/Arthritis Foundation guidelines for treatment of JIA were published [34]. These were unfortunately not available for consideration during our study; however, none of the KPIs currently included in our set addresses treatment at this time and it is unlikely based on the KPIs considered by panelists that the guidelines would have altered the final resulting set. In the future, the updated guidelines should be considered when developing additional JIA treatment KPIs. Lastly, there exist a number of challenges for the development of outcome measures including but not limited to outcome attribution, risk adjustment, and defining the period of risk (reviewed in detail by Suter et al. [35]). We therefore propose that this set represent a starter set for data collection and monitoring as the processes required for documenting important patient outcomes including pain, disease activity, and function are included. Future projects would work to define appropriate outcome measures and benchmarks for care.

It should be noted that while all KPIs considered for inclusion in the framework were rated highly by our panel in terms of importance and priority for measurement, there were significant concerns around the feasibility of measurement of some KPIs, which lead to their exclusion. This should not be interpreted as a lack of importance of these measures necessarily; however, this highlights that improved methods of data collection could result in the addition of some of these measures to the framework at a future date. For example, while critical to high-quality JIA care, uveitis screening documentation was felt to be a challenge in many centers, which could have led to falsely low measure reporting and hence lower feasibility ratings by our panelists. Future work should therefore focus on improving reliable ways to track screening in electronic records. There was also skepticism about how patient experience and quality of life questionnaires could be used to improve patient care and/or health leading to the exclusion of measures relating to these concepts. A lack of standardized questionnaires in these domains was also highlighted, as were concerns about length of existing questionnaires and burden to patient and families receiving care and collection of such questionnaires is not currently routinely completed in practice. Further research in the area of patient experience and quality of life can be used to drive clinical improvements and may help inform future performance measurement in this area.

## Conclusions

Our KPI framework was developed building upon existing frameworks in the USA and the UK and highlights important processes of JIA care with measures adapted to the Canadian context. The feasibility of capturing these measures will be examined in available data sources, including the data collected prospectively as part of a national study (UCAN CURE: Precision Decisions in Childhood Arthritis). Ultimately, these measures may also inform routine clinical practice and quality reporting. Importantly, the collection of these process KPIs systematically will allow us to evaluate the impact on patient outcomes and inform the development of meaningful outcome KPIs in the future.

## Supplementary information

**Additional file 1.** Select websites searched for grey literature review. Sources and website links for sites searched for grey literature review.

**Additional file 2.** Select guidelines or standards of care or recommendations endorsed by various medical societies. Sources and links/references for guidelines, standards of care and recommendations endorsed by various medical societies.

**Additional file 3.** Summary of proposed Key Performance Indicators (KPIs), modifications and rationale. Summary of modifications to KPIs and rationale by the working group or Delphi panel.

**Additional file 4.** Gaps in measures according to guidelines, standards of care and recommendations for JIA treatment. Gaps in measure that were identified when comparing the measures found through the systematic review to published JIA guidelines, standards of care and recommendations.

**Additional file 5.** Final set of KPIs: descriptions and reporting. Full descriptions and reporting details for the final set of KPIs. Is an extension of Table 4 in manuscript.

## Data Availability

The datasets used and/or analyzed during the current study are available from the corresponding author on reasonable request.

## Abbreviations

AAC: Arthritis Alliance of Canada
ACR: American College of Rheumatology
Anti-TNF: Anti-tumor necrosis factor inhibitors
BSPAR: British Society for Paediatric and Adolescent Rheumatology
CHAQ: Childhood Health Assessment Questionnaire
cJADAS: Clinical Juvenile Arthritis Disease Activity Score
DMARDs: Disease-modifying anti-rheumatic drugs
IOM: Institute of Medicine
IPRAS: Interpercentile Range Adjusted for Symmetry
JIA: Juvenile idiopathic arthritis
KPIs: Key performance indicators
PR-COIN: Pediatric Rheumatology Care and Outcomes Improvement Network
RAND/UCLA: RAND Corporation/University of California Los Angeles
UCAN: Understanding Childhood Arthritis Network
