# Supplementary material for: A Canadian evaluation framework for quality improvement in childhood arthritis: key performance indicators of the process of care
Source: Arthritis Res Ther. 2020 Mar 19;22:53. doi: 10.1186/s13075-020-02151-w (PMC7083048; doi:10.1186/s13075-020-02151-w)
Supplement: Supplementary file 1 — Additional file 1. Select websites searched for grey literature review. Sources and website links for sites searched for grey literature review. [file 13075_2020_2151_MOESM1_ESM.docx]

**Additional file 1: Methods for Systematic Literature Review Update**

A systematic review of quality measures for inflammatory arthritis by Cooper et al. [1] was updated for the present study. The original search by Cooper et al. was executed on October 23^rd^, 2016 so for the updated search, the years of the search included 2016 to 2019. The senior author of the original study (Barber) had access to the original search strategies. These were re-run using the quality measure search terms and the juvenile idiopathic arthritis (JIA) search terms on January 14^th^, 2019. The search was run in 3 databases including Medline, CINHAL, and Embase. The search strategy for Medline is shown below. In addition, a grey literature search of relevant websites was conducted for unpublished quality measures (see table of websites searched below). The original systematic review inclusion and exclusion criteria were used [1]: “Quality measures were included if they were in English and identified an element of inflammatory arthritis care (in this case JIA care), and the method of development of the quality measures was available. Checklists or quality measures with no description of how they were selected and/or developed were excluded, as were quality standards (they lacked specific numerators and denominators and were not measurable). If the development of the quality measure was not outlined on a potentially eligible measure set, attempts were made to contact the author to determine whether the development strategy was available. Studies describing the use of quality measures, and not the development of the measures, were excluded.” Articles were selected for inclusion by 2 authors (CB and TP). The results of the updated search are shown in a flow diagram below.

Medline JIA search run Jan 14^th^, 2019

| 1. exp Quality Indicators, Health Care/ |
| --- |
| 2. exp Quality Assurance, Health Care/ |
| 3. exp Quality Improvement/ |
| 4. exp "Standard of Care"/ |
| 5. exp Total Quality Management/ |
| 6. exp Quality Control/ |
| 7. exp peer review, health care/ |
| 8. (Quality adj Assurance).tw. |
| 9. (Quality adj of adj Health adj Care).tw. |
| 10. (quality adj improvement$).tw. |
| 11. (quality adj indicator$).tw. |
| 12. (quality adj criterion$).tw. |
| 13. (quality adj measure$).tw. |
| 14. (performance adj indicator$).tw. |
| 15. (performance adj measure$).tw. |
| 16. (outcome adj indicator$).tw. |
| 17. (process adj assessment$).tw. |
| 18. (structure adj indicator$).tw. |
| 19. (quality adj parameter$).tw. |
| 20. (process adj parameter$).tw. |
| 21. (structure adj parameter$).tw. |
| 22. (outcome adj parameter$).tw. |
| 23. (performance adj parameter$).tw. |
| 24. (quality adj assessment$).tw. |
| 25. (clinical adj indicator$).tw. |
| 26. clinical audit/ |
| 27. (clinical adj audit).tw. |
| 28. (medical adj audit).tw. |
| 29. medical audit/ |
| 30. benchmarking/ |
| 31. benchmarking.tw. |
| 32. (standard$ adj of adj care).tw. |
| 33. (total adj quality adj management).tw. |
| 34. (quality adj control).tw. |
| 35. or/1-34 |
| 36. (inflammatory adj arthr$).tw. |
| 37. (early adj arthr$).tw. |
| 38. (chronic adj arthr$).tw. |
| 39. oligoarth$.tw. |
| 40. Arthritis, Juvenile/ |
| 41. (juvenile$ adj3 arthr$).tw. |
| 42. (juvenile$ adj3 polyarthr$).tw. |
| 43. (child$ adj3 arthr$).tw. |
| 44. (still$ adj disease).tw. |
| 45. (enthesitis adj2 arthr$).tw. |
| 46. JRA.tw. |
| 47. JIA.tw. |
| 48. or/36-47 |
| 49. 35 and 48 |
| 50. limit 49 to english language |
| 51. limit 50 to yr="2016-Current" |

**Grey Literature Search**

| **Source** | **Website** |
| --- | --- |
| **North American sources** | |
| American college of rheumatology (ACR) | www.rheumatology.org |
| Canadian Institute for Health Information (CIHI) Indicator Library | indicatorlibrary.cihi.ca/display/HSPIL/Indicator+Library?desktop=true |
| Childhood Arthritis and Rheumatology Research Alliance (CARRA) | www.carragroup.org |
| National Quality Measures Clearinghouse (Agency for Health Care Research [AHRQ]) | www.qualitymeasures.ahrq.gov |
| National Quality Forum | www.qualityforum.org/Home.aspx |
| Pediatric rheumatology care & outcomes improvement network (PR-COIN) | www.pr-coin.org |
| **European sources** | |
| National Institute for Health and Clinical Excellence (NICE) | [www.nice.org.uk/aboutnice/qof/indicators.jsp](http://www.nice.org.uk/aboutnice/qof/indicators.jsp) |
| British Society for Paediatric and adolescent Rheumatology (BSPAR) | www.bspar.org.uk/https://www.rheumatology.org.uk |
| Paediatric Rheumatology International Trials Organization (PRINTO): Single Hub and access point for paediatric rheumatology in Europe (SHARE) | https://www.printo.it/share |
| Paediatric Rheumatology European Society (PReS) | www.pres.eu |
| Scottish Intercollegiate guidelines Network (SIGN) | https://www.sign.ac.uk/ |
| SPARN (Scottish Pediatric and adolescent rheumatology network) | https://www.sparn.scot.nhs.uk/ |


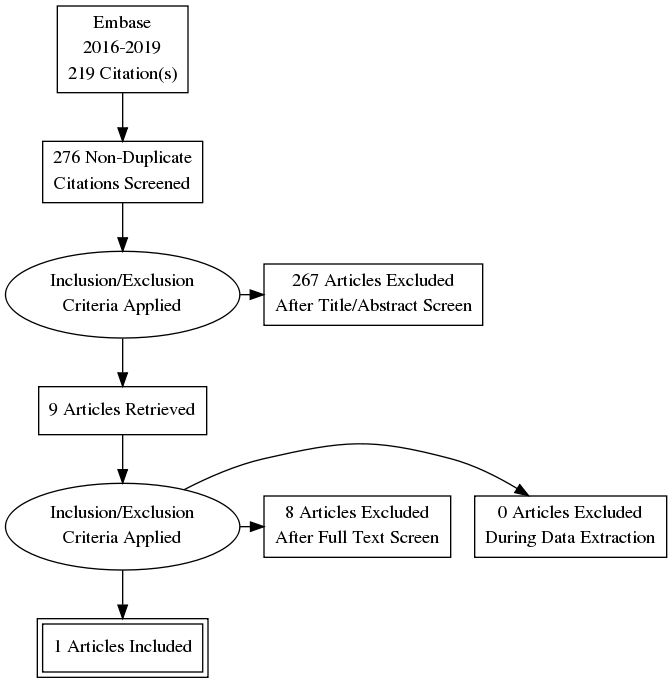


Figure 1. Flow diagram of update to systematic review of quality measures for juvenile idiopathic arthritis (JIA)

1. Cooper M, Rouhi A, Barber CEH. A Systematic Review of Quality Measures for Inflammatory Arthritis. J Rheumatol. 2018;45(2):274-83.
