# Supplementary material for: A Canadian evaluation framework for quality improvement in childhood arthritis: key performance indicators of the process of care
Source: Arthritis Res Ther. 2020 Mar 19;22:53. doi: 10.1186/s13075-020-02151-w (PMC7083048; doi:10.1186/s13075-020-02151-w)
Supplement: Supplementary file 2 — Additional file 2. Select guidelines or standards of care or recommendations endorsed by various medical societies. Sources and links/references for guidelines, standards of care and recommendations endorsed by various medical societies. [file 13075_2020_2151_MOESM2_ESM.docx]

**Additional file 2: Select guidelines or standards of care or recommendations endorsed by various medical societies**

| **Source** | **Reference** |
| --- | --- |
| **Guidelines or standards of care** | |
| American College of Rheumatology (ACR) | - Beukelman T, Patkar NM, Saag KG, Tolleson-Rinehart S, Cron RQ, DeWitt EM, et al. 2011 American College of Rheumatology recommendations for the treatment of juvenile idiopathic arthritis: Initiation and safety monitoring of therapeutic agents for the treatment of arthritis and systemic features. Arthritis Care & Research. 2011;63(4):465-82. - Ringold S, Weiss PF, Beukelman T, DeWitt EM, Ilowite NT, Kimura Y, et al. 2013 update of the 2011 American College of Rheumatology recommendations for the treatment of juvenile idiopathic arthritis: recommendations for the medical therapy of children with systemic juvenile idiopathic arthritis and tuberculosis screening among children receiving biologic medications. Arthritis Rheum. 2013;65(10):2499-512. |
| Canadian Rheumatology Association (CRA) | - Cellucci T, Guzman J, Petty RE, Batthish M, Benseler SM, Ellsworth JE, et al. Management of Juvenile Idiopathic Arthritis 2015: A Position Statement from the Pediatric Committee of the Canadian Rheumatology Association. The Journal of Rheumatology. 2016;43(10):1773-6. |
| British Society of Paediatric and Adolescent Rheumatology (BSPAR) | - Davies K, Cleary G, Foster H, Hutchinson E, Baildam E; British Society of Paediatric and Adolescent Rheumatology. BSPAR Standards of Care for children and young people with juvenile idiopathic arthritis. Rheumatology 2010;49:1406-8. |
| The Royal Australian College of General Practitioners (RACGP) | - The Royal Australian College of General Practitioners. Recommendations for the diagnosis and management of juvenile idiopathic arthritis 2009.https://www.racgp.org.au/   FSDEDEV/media/documents/Clinical%20Resources/  Guidelines/Joint%20replacement/Juvenile-idiopathic-arthritis-recommendations.pdf. Accessed February 2019. |
| Pediatric Rheumatology Association of Japan | - Okamoto N, Yokota S, Takei S, Okura Y, Kubota T, Shimizu M, et al. Clinical practice guidance for juvenile idiopathic arthritis (JIA) 2018. Modern Rheumatology. 2019;29(1):41-59. |
| German Society for Pediatric Rheumatology (GKJR) | - Dueckers G, Guellac N, Arbogast M, Dannecker G, Foeldvari I, Frosch M, et al. Evidence and consensus based GKJR guidelines for the treatment of juvenile idiopathic arthritis. Clin Immunol. 2012;142(2):176-93. |
| **Recommendations** | |
| Ravelli A, Consolaro A, Horneff G, Laxer RM, Lovell DJ, Wulffraat NM, et al. | - Ravelli A, Consolaro A, Horneff G, Laxer RM, Lovell DJ, Wulffraat NM, et al. Treating juvenile idiopathic arthritis to target: recommendations of an international task force. Ann Rheum Dis. 2018;77(6):819-28. |
| Paediatric Rheumatology International Trials Organization (PRINTO)/ Single Hub and Access point for Paediatric Rheumatology in Europe (SHARE) | - Constantin T, Foeldvari I, Anton J, de Boer J, Czitrom-Guillaume S, Edelsten C, et al. Consensus-based recommendations for the management of uveitis associated with juvenile idiopathic arthritis: the SHARE initiative. Annals of the rheumatic diseases. 2018;77(8):1107-17. |
| Ferrara G, Mastrangelo G, Barone P, La Torre F, Martino S, Pappagallo G, et al. | - Ferrara G, Mastrangelo G, Barone P, La Torre F, Martino S, Pappagallo G, et al. Methotrexate in juvenile idiopathic arthritis: advice and recommendations from the MARAJIA expert consensus meeting. Pediatric Rheumatology. 2018;16(1):46. |
| European League Against Rheumatism (EULAR)/ Paediatric Rheumatology European Society (PReS) | - Foster HE, Minden K, Clemente D, Leon L, McDonagh JE, Kamphuis S, et al. EULAR/PReS standards and recommendations for the transitional care of young people with juvenile-onset rheumatic diseases. Annals of the Rheumatic Diseases. 2017;76(4):639-46. |
