# Supplementary material for: A Canadian evaluation framework for quality improvement in childhood arthritis: key performance indicators of the process of care
Source: Arthritis Res Ther. 2020 Mar 19;22:53. doi: 10.1186/s13075-020-02151-w (PMC7083048; doi:10.1186/s13075-020-02151-w)
Supplement: Supplementary file 3 — Additional file 3. Summary of proposed Key Performance Indicators (KPIs), modifications and rationale. Summary of modifications to KPIs and rationale by the working group or Delphi panel. [file 13075_2020_2151_MOESM3_ESM.docx]

**Additional file 3: Summary of proposed Key Performance Indicators, modifications and rationale**

| **KPI name** | **Original KPI description** | **Proposed modifications by working group (WG) or Delphi panel (DP)^*^** | **Rationale for recommendation or modification** | **Final KPI description** |
| --- | --- | --- | --- | --- |
| **Indicator 1**: Assessment of arthritis-related pain | Pain should be assessed in all patients at the first visit and at each subsequent visit that occur at least 7 days apart  [1]. | No modifications. | N/A | Pain should be assessed in all patients at the first visit and at each subsequent visit that occur at least 7 days apart. |
| **Indicator 2**: Rheumatological joint count | A full joint count of all 75 joints should be done on all patients at the first visit and at 6-month intervals [1]. | A full joint count of all 75 joints should be done on all patients at the first visit and at every routine clinic visit (WG).  A full joint count should be done on all patients at the first visit and at every routine clinic visit (DP). | The interval for assessment was changed to be consistent with Canadian clinical practice (WG).  There was consensus among panel members that a full joint count of all 75 joints would be difficult to document. Therefore, the KPI was simplified to exclude individual documentation of all 75 joints (DP). | A joint count should be done on all patients at the first visit and at every routine clinic visit. |
| **Indicator 3**:  Physician’s Global Assessment of disease activity | A PGA should be completed on all patients at the initial visit and at each subsequent visit [1]. | No modifications. | N/A | A PGA should be completed on all patients at the initial visit and at each subsequent visit. |
| **Indicator 4**: Assessment of functional ability | All patients should receive an assessment of functional ability at the initial visit and at a minimum of 6-month interval thereafter [1]. | All patients should receive an assessment of functional ability at the initial visit and at every routine clinic visit (DP). | The interval for assessment was changed to be consistent with Canadian clinical practice (DP). | All patients should receive an assessment of functional ability at the initial visit and at every routine clinic visit. |
| **Indicator 5:** Assessment of functional ability using the CHAQ | All patients should receive an assessment of functional ability using the CHAQ at every routine clinic visit. | New proposed indicator (WG). | This indicator specifies the use of the CHAQ. The CHAQ includes both the patient/ parent PGA and is routinely collected in clinic (WG). | Excluded because it did not fulfill criteria for panel consensus. |
| **Indicator 6**:  Composite disease activity measurement | Percentage of patients 16 years and younger with a diagnosis of JIA and >=50% of total number of outpatient encounters in the measurement year with assessment of disease activity using a standardized measure. | New proposed indicator (WG).  Percentage of patients 16 years and younger with a diagnosis of JIA with an assessment of disease activity using the cJADAS at every routine clinic visit (DP). | The use of a single composite measure will streamline the data collection process and ensure that data sources are consistent among various pediatric centres across Canada (WG).  The cJADAS was recommended to eliminate the need for laboratory services in the assessment of disease activity. Frequency of assessment was changed to be consistent with “treat to target” (DP). | Percentage of patients 16 years and younger with a diagnosis of JIA with an assessment of disease activity using the cJADAS at every routine clinic visit. |
| **Indicator 7**: Tuberculosis screening | All patients with JIA will undergo TB screening no longer than 3 months prior to the start of biologic therapy and yearly thereafter as long as the patient remains on biologic therapy [1]. | All patients with JIA will undergo TB screening no longer than 3 months prior to the start of any biologic therapy (WG).  All patients with JIA, with a consideration of risk factors, will undergo TB screening within 12 months prior to receiving a first course of therapy using a biologic DMARD (DP). | The interval for assessment was changed to be consistent with Canadian clinical practice (WG).  The interval of assessment for TB screening was modified again to be consistent with guidelines. The test is not routinely done if the patient is tolerating the medication and it is important to consider risk factors for TB on a case by case basis (DP). | All patients with JIA, with a consideration of risk factors, will undergo TB screening within 12 months prior to receiving a first course of therapy using a biologic DMARD. |
| **Indicator 8**:  Laboratory monitoring for DMARDs | All JIA patients receiving DMARD or biologic therapy will be monitored for toxicity by clinical laboratory methods. The minimal frequency monitoring for DMARDs is every 2-4 weeks for the first 3 months of therapy, every 8-12 weeks after 3-6 months, and every 12 weeks after 6 months of therapy [1]. | All JIA patients receiving methotrexate and leflunomide will be monitored for toxicity by clinical laboratory methods. The minimal frequency of laboratory monitoring is at least monthly for the first 3 months of therapy and every 3 months thereafter (WG).  **All JIA patients receiving methotrexate or leflunomide will be monitored for toxicity by clinical laboratory methods. The minimal frequency of laboratory monitoring is** 1 month after the start of therapy and every 3-4 months thereafter (DP). | Methotrexate and leflunomide were chosen because these are common DMARDs used to treat JIA. The interval for assessment was modified to be consistent with Canadian clinical practice (WG).  The interval for assessment was further modified to be consistent with Canadian clinical practice (DP). | **All JIA patients receiving methotrexate or leflunomide will be monitored for toxicity by clinical laboratory methods. The minimal frequency of laboratory monitoring is** 1 month after the start of therapy and every 3-4 months thereafter. |
| **Indicator 9**:  Waiting times for rheumatologist consultation for patients with new onset JIA | The number of days that patients waited between the date the initial referral was received and the date of consultation with a rheumatologist for patients with new onset JIA where the diagnosis of JIA is made or confirmed by a pediatric rheumatologist [2]. | No modifications. | N/A | The number of days that patients waited between the date the initial referral was received and the date of consultation with a rheumatologist for patients with new onset JIA where the diagnosis of JIA is made or confirmed by a pediatric rheumatologist. |
| **Indicator 10**:  Percentage of patients with JIA seen by a rheumatologist | The percentage of patients with new onset JIA with at least one visit to a pediatric rheumatologist in the first year of diagnosis [2]. | No modifications. | N/A | The percentage of patients with new onset JIA with at least one visit to a pediatric rheumatologist in the first year of diagnosis. |
| **Indicator 11**:  Percentage of patients seen in follow-up by a pediatric rheumatologist | The percentage of patients with a diagnosis of JIA under the care of a pediatric rheumatologist seen in follow-up by a pediatric rheumatologist at least once per year [2]. | No modifications. | N/A | The percentage of patients with a diagnosis of JIA under the care of a pediatric rheumatologist seen in follow-up by a pediatric rheumatologist at least once per year. |
| **Indicator 12**:  Median time from the patient's first clinic visit to the date of their first uveitis screening | What is the median time from the patient’s first clinic visit to the date of their first uveitis screening with an appropriate pediatric ophthalmic specialists, for patients with different ILAR sub-types? [3] | What is the median time from the patient’s first clinic visit to the date of their first uveitis screening with an appropriate eye specialists? (WG)  What is the median time from the patient's first clinic visit to the date of their first uveitis screening? (DP) | The KPI was broadened to include all health care professionals with appropriate training to conduct eye screening (e.g., ophthalmologists or optometrists) (WG)  Panelists agreed that it would be difficult to ascertain who is qualified to conduct uveitis screening and “appropriate eye specialists” was removed (DP). | Excluded because it did not fulfill criteria for panel consensus. |
| **Indicator 13**: Assessment of Health-Related Quality of Life (HRQOL) | All patients should receive an assessment of HRQOL at the initial visit and at 6-month intervals [2]. | All patients should receive an assessment of HRQOL at the initial visit and at every routine clinic visit (DP). | The interval for assessment was modified to account for variation in data collection (DP). | Excluded because it did not fulfill criteria for panel consensus. |
| **Indicator 14**:  Assessment of patients/parent satisfaction with care | Patients and/or parents should be assessed for their level of satisfaction with the quality of care provided to them/their child. The first assessment should be done no longer than 1 year after the initial visit, and annually thereafter [1]. | No modifications. | N/A | Excluded because it did not fulfill criteria for panel consensus. |

N/A: Not applicable; KPI: Key Performance Indicator; PGA: Physician’s Global Assessment; cJADAS: Clinical Juvenile Arthritis Disease Activity Score; DMARDs: Disease modifying anti-rheumatic drugs; TB: Tuberculosis screening.

References

1. Lovell DJ, Passo MH, Beukelman T, Bowyer SL, Gottlieb BS, Henrickson M, et al. Measuring process of arthritis care: a proposed set of quality measures for the process of care in juvenile idiopathic arthritis. Arthritis Care & Research. 2011;63(1):10-6.

2. Barber CEH, Marshall DA, Mosher DP, Akhavan P, Tucker L, Houghton K, et al. Development of System-level Performance Measures for Evaluation of Models of Care for Inflammatory Arthritis in Canada. The Journal of Rheumatology. 2016;43(3):530-40.

3. McErlane F, Foster HE, Armitt G, Bailey K, Cobb J, Davidson JE, et al. Development of a national audit tool for juvenile idiopathic arthritis: a BSPAR project funded by the Health Care Quality Improvement Partnership. Rheumatology (Oxford). 2018;57(1):140-51.
