# Supplementary material for: A Canadian evaluation framework for quality improvement in childhood arthritis: key performance indicators of the process of care
Source: Arthritis Res Ther. 2020 Mar 19;22:53. doi: 10.1186/s13075-020-02151-w (PMC7083048; doi:10.1186/s13075-020-02151-w)
Supplement: Supplementary file 4 — Additional file 4. Gaps in measures according to guidelines, standards of care and recommendations for JIA treatment. Gaps in measure that were identified when comparing the measures found through the systematic review to published JIA guidelines, standards of care and recommendations. [file 13075_2020_2151_MOESM4_ESM.docx]

**Additional file 4: Gaps in measures according to guidelines, standards of care and recommendations for JIA treatment**

| **Measure or consensus statement or standard** | **Reference** |
| --- | --- |
| **Non-drug-based therapies** | |
| First assessment by pediatric rheumatology physiotherapist ≤8 weeks from the time of referral or no longer than 2 weeks after joint injections. | - Davies K, Cleary G, Foster H, Hutchinson E, Baildam E; British Society of Paediatric and Adolescent Rheumatology. BSPAR Standards of Care for children and young people with juvenile idiopathic arthritis. Rheumatology 2010; 49:1406-8. |
| Structured treatment by a properly trained physiotherapist or occupational therapist in combination with drug-based therapy and instructions for disease adopted, self-sufficient daily exercise sessions are recommended to keep and to improve joint mobility. | - Dueckers G, Guellac N, Arbogast M, Dannecker G, Foeldvari I, Frosch M, et al. Evidence and consensus based GKJR guidelines for the treatment of juvenile idiopathic arthritis. Clin Immunol. 2012;142(2):176-93.   *Also mentioned in:*   - Cellucci T, Guzman J, Petty RE, Batthish M, Benseler SM, Ellsworth JE, et al. Management of Juvenile Idiopathic Arthritis 2015: A Position Statement from the Pediatric Committee of the Canadian Rheumatology Association. The Journal of Rheumatology. 2016;43(10):1773-6. - The Royal Australian College of General Practitioners. Recommendations for the diagnosis and management of juvenile idiopathic arthritis 2009. https://www.racgp.org.au/FSDEDEV/media/documents/Clinical%20Resources/   Guidelines/Joint%20replacement/Juvenile-idiopathic-arthritis-recommendations.pdf. Accessed February 2019. |
| Exercise training is recommended depending on the extent of inflammation, number of affected joints and global disease activity. Sports with minor stress on joints is favorable. | - Dueckers G, Guellac N, Arbogast M, Dannecker G, Foeldvari I, Frosch M, et al. Evidence and consensus based GKJR guidelines for the treatment of juvenile idiopathic arthritis. Clin Immunol. 2012;142(2):176-93.   *Also mentioned in:*   - Cellucci T, Guzman J, Petty RE, Batthish M, Benseler SM, Ellsworth JE, et al. Management of Juvenile Idiopathic Arthritis 2015: A Position Statement from the Pediatric Committee of the Canadian Rheumatology Association. The Journal of Rheumatology. 2016;43(10):1773-6. |
| **Transitional care** | |
| All young people with JIA should have a planned, coordinated transition from the pediatric to the adult service. | - Davies K, Cleary G, Foster H, Hutchinson E, Baildam E; British Society of Paediatric and Adolescent Rheumatology. BSPAR Standards of Care for children and young people with juvenile idiopathic arthritis. Rheumatology 2010; 49:1406-8.   *Also mentioned in:*   - Foster HE, Minden K, Clemente D, Leon L, McDonagh JE, Kamphuis S, et al. EULAR/PReS standards and recommendations for the transitional care of young people with juvenile-onset rheumatic diseases. Annals of the Rheumatic Diseases. 2017;76(4):639-46. |
| **Therapy assessment and safety monitoring** | |
| Response to therapy should be assessed after 3 months of therapy and reassessed every 3 months while treatment continues. | - National Health Service England. Clinical Commissioning Policy Statement: Biologic Therapies for the treatment of Juvenile Idiopathic Arthritis (JIA) 2015. https://www.england.nhs.uk/wp-content/uploads/2018/08/Biologic-therapies-for-the-treatment-of-juvenile-idiopathic-arthritis.pdf |
| All children who commence treatment with a biologic should be offered the option of enrolling in the appropriate long-term national Registries. | - National Health Service England. Clinical Commissioning Policy Statement: Biologic Therapies for the treatment of Juvenile Idiopathic Arthritis (JIA) 2015. https://www.england.nhs.uk/wp-content/uploads/2018/08/Biologic-therapies-for-the-treatment-of-juvenile-idiopathic-arthritis.pdf |
| In all patients, at least a 50% improvement in disease activity should be reached within 3 months and the target within 6 months. | - Ravelli A, Consolaro A, Horneff G, Laxer RM, Lovell DJ, Wulffraat NM, et al. Treating juvenile idiopathic arthritis to target: recommendations of an international task force. Ann Rheum Dis. 2018;77(6):819-28. |
| Measurement of serum creatinine, urinalysis, complete blood cell count, and liver enzymes was recommended prior to or soon after the initiation of treatment with routine NSAIDs. Periodic repeat measurements of serum creatinine, urinalysis, complete blood cell count, and liver enzymes were recommended approximately twice yearly for patients receiving chronic daily NSAIDs and approximately once yearly for patients receiving NSAIDs routinely (e.g., 3 to 4 days per week). | - Beukelman T, Patkar NM, Saag KG, Tolleson-Rinehart S, Cron RQ, DeWitt EM, et al. 2011 American College of Rheumatology recommendations for the treatment of juvenile idiopathic arthritis: Initiation and safety monitoring of therapeutic agents for the treatment of arthritis and systemic features. Arthritis Care & Research. 2011;63(4):465-82. |
| Antibody testing for infection with hepatitis B or hepatitis C prior to initiating methotrexate or TNFα inhibitors was recommended for patients with risk factors for infection. | - Beukelman T, Patkar NM, Saag KG, Tolleson-Rinehart S, Cron RQ, DeWitt EM, et al. 2011 American College of Rheumatology recommendations for the treatment of juvenile idiopathic arthritis: Initiation and safety monitoring of therapeutic agents for the treatment of arthritis and systemic features. Arthritis Care & Research. 2011;63(4):465-82. |
| **Vaccinations for communicable diseases** |  |
| Quality measures examining vaccinations (e.g., influenza and pneumococcal) for those with rheumatoid arthritis (RA) have been previously developed. These quality measures were based on RA guidelines and adapted for RA using the Canadian Institute of Health Information quality measures for the general population. | *Measure not specifically stated in JIA guidelines but are recommended by RA guidelines.*   - Singh JA, Saag KG, Bridges SL, Jr., Akl EA, Bannuru RR, Sullivan MC, et al. 2015 American College of Rheumatology guideline for the treatment of rheumatoid arthritis. Arthritis Rheumatol 2016;68:1-26. - Bombardier C, Hazlewood GS, Akhavan P, Schieir O, Dooley A, Haraoui B, et al. Canadian Rheumatology Association recommendations for the pharmacological management of rheumatoid arthritis with traditional and biologic disease-modifying antirheumatic drugs: part II safety. The Journal of rheumatology. 2012;39(8):1583-602. |
