# Supplementary material for: A Canadian evaluation framework for quality improvement in childhood arthritis: key performance indicators of the process of care
Source: Arthritis Res Ther. 2020 Mar 19;22:53. doi: 10.1186/s13075-020-02151-w (PMC7083048; doi:10.1186/s13075-020-02151-w)
Supplement: Supplementary file 5 — Additional file 5. Final set of KPIs: descriptions and reporting. Full descriptions and reporting details for the final set of KPIs. Is an extension of Table 4 in manuscript. [file 13075_2020_2151_MOESM5_ESM.docx]

**Additional file 5: Final set of Key Performance Indicators: descriptions and reporting**

| **KPI name** | **Final descriptions** | **Reporting during measurement period** |
| --- | --- | --- |
| Assessment of arthritis-related pain | Pain should be assessed in all patients at the first visit and at each subsequent visit that occur at least 7 days apart. | The percentage of patients assessed for pain at the first visit and each subsequent visit using any validated age-appropriate tool to measure average pain. |
| Rheumatological joint count | A joint count should be done on all patients at the first visit and at every routine clinic visit. | The percentage of patients where a joint count was conducted on the first visit and each subsequent visit using a validated tool. |
| Physician’s Global Assessment of disease activity | A PGA should be completed on all patients at the initial visit and at each subsequent visit. | The percentage of patients assessed for a PGA using any validated tool at the first visit and at each subsequent visit. |
| Assessment of functional ability | All patients should receive an assessment of functional ability at the initial visit and at every routine clinic visit. | The percentage of patients assessed for functional ability using any validated tool at the first visit and at every routine clinic visit. |
| Composite disease activity measurement | Percentage of patients 16 years and younger with a diagnosis of JIA with an assessment of disease activity cJADAS at every routine clinic visit. | The percentage of patients in the measurement period with an assessment of disease activity using the cJADAS. |
| Tuberculosis screening | All patients with JIA, with a consideration of risk factors, will undergo TB screening within 12 months prior to receiving a first course of therapy using a biologic DMARD. | The percentage of patients screened for TB within 12 months prior to receiving a first course of therapy using a biologic DMARD. |
| Laboratory monitoring for DMARDS | All JIA patients receiving methotrexate or leflunomide will be monitored for toxicity by clinical laboratory methods. The minimal frequency of laboratory monitoring is 1 month after the start of therapy, and every 3-4 months thereafter. | The percentage of patients who received methotrexate and leflunomide and monitored for toxicity by clinical laboratory methods. |
| Waiting times for rheumatologist consultation for patients with new onset JIA | The number of days that patients waited, between the date the initial referral was received and the date of consultation with a rheumatologist for patients with new onset JIA where the diagnosis of JIA is made or confirmed by a pediatric rheumatologist. | The 50^th^ and 90^th^ percentile waiting times for rheumatologic consultation. |
| Percentage of patients with JIA seen by a rheumatologist | The percentage of patients with new onset JIA with at least one visit to a pediatric rheumatologist in the first year of diagnosis. | The percentage of patients with new onset JIA (incident JIA) with at least 1 visit to a pediatric rheumatologist in the first year of diagnosis. |
| Percentage of patients seen in yearly follow-up by a pediatric rheumatologist | The percentage of patients with a diagnosis of JIA under the care of a pediatric rheumatologist seen in follow-up by a pediatric rheumatologist at least once per year. | The percentage of patients with JIA seen by their pediatric rheumatologist at least once every year. |

KPI: Key Performance Indicator; PGA: Physician’s Global Assessment; cJADAS: Clinical Juvenile Arthritis Disease Activity Score; DMARDs: Disease modifying anti-rheumatic drugs; TB: Tuberculosis screening.
